# Impact of Annual Dry Weight Changes on Mortality and Cardiovascular Outcomes in Patients Undergoing Haemodialysis

**DOI:** 10.1002/jcsm.70100

**Published:** 2025-10-14

**Authors:** Yoosun Joo, Jihoon Park, Yang‐Gyun Kim, Sang‐Ho Lee, Ju‐Young Moon, Soo‐Young Yoon, Hyeon Seok Hwang, Jihyun Baek, Dong‐Young Lee, Gang Jee Ko, Min‐Jeong Lee, Seok Hui Kang, Su Woong Jung

**Affiliations:** ^1^ Division of Nephrology, Department of Internal Medicine Kyung Hee University Hospital at Gangdong Seoul Republic of Korea; ^2^ Department of Medicine, Graduate School Kyung Hee University Seoul Republic of Korea; ^3^ Division of Nephrology, Department of Internal Medicine, College of Medicine Kyung Hee University Seoul Republic of Korea; ^4^ Division of Nephrology, Department of Internal Medicine, Kyung Hee University Hospital Kyung Hee University College of Medicine Seoul Republic of Korea; ^5^ Division of Nephrology, Department of Internal Medicine CHA University Bundang Medical Center Seongnam Republic of Korea; ^6^ Division of Nephrology, Department of Internal Medicine VHS Medical Center Seoul Republic of Korea; ^7^ Division of Nephrology, Department of Internal Medicine Korea University College of Medicine Seoul Republic of Korea; ^8^ Division of Nephrology, Department of Internal Medicine Ajou University Hospital Suwon Republic of Korea; ^9^ Division of Nephrology, Department of Internal Medicine Yeungnam University College of Medicine Daegu Republic of Korea

**Keywords:** cardiovascular event, dry weight change, haemodialysismortality

## Abstract

**Background:**

While obesity confers a survival advantage, weight loss adversely affects the survival of patients undergoing haemodialysis. However, given the limited information regarding its long‐term effects on mortality and cardiovascular events, the health benefits of weight gain remain uncertain, particularly in Asian patients undergoing haemodialysis.

**Methods:**

In a prospective multicentre cohort of patients undergoing haemodialysis in South Korea, patients whose dry weight was recorded at baseline and after 1 year were analysed. Patients were stratified into five groups according to annual dry weight change: stable (−2.0% to 1.9%, *n* = 245), mild (2.0% to 6.9%, *n* = 92) and moderate (≥ 7.0%, *n* = 20) dry weight gain and mild (−5.0% to −2.1%, *n* = 91) and moderate (< −5.0%, *n* = 77) dry weight loss. The associations of annual dry weight change with physical function and health‐related quality of life were examined using cross‐sectional analysis. The impact of annual dry weight changes on all‐cause mortality and a composite of major adverse cardiovascular events (MACEs), defined as myocardial infarction, unstable angina, ischaemic stroke and peripheral artery disease requiring revascularization, was assessed in a longitudinal cohort of 525 individuals.

**Results:**

In cross‐sectional analysis, patients with diminished physical ability had a higher frequency of dry weight fluctuations. In longitudinal analysis, the mean age of the study participants was 59.9 years, and 62.3% were men. During a median follow‐up of 3.1 years, death and MACE occurred in 105 (20.0%) and 31 (5.9%) patients, respectively. The risk of all‐cause mortality was higher in patients with moderate dry weight gain or loss than in those with stable dry weight (adjusted hazard ratio [aHR] for moderate weight gain, 2.22; 95% confidence interval [CI], 0.96**–**5.13; *p* = 0.06; and aHR for moderate weight loss, 1.78; 95% CI, 1.07**–**2.95; *p* = 0.03). The risk of MACE was significantly higher in patients with weight gain (including mild and moderate) than in those with a stable dry weight (aHR, 3.02; 95% CI, 1.32**–**6.88; *p* = 0.009). Specifically, the increased risk of all‐cause mortality attributable to moderate dry weight gain was limited to patients with obesity, whereas that for moderate dry weight loss was limited to patients with a normal body mass index.

**Conclusion:**

Moderate weight gain and loss were differentially associated with lower survival among patients undergoing haemodialysis, with the former in patients with obesity and the latter in normal‐weight patients. Particularly, dry weight gain increased the risk of cardiovascular events.

## 1 Introduction

End‐stage kidney disease (ESKD) treated with dialysis is a global burden, with its incidence increasing by 43.1% since 1990 [1]. According to a recent Dialysis Outcomes and Practice Patterns Study, patient survival is improving globally and is attributable to improvements in specific facility practices, especially by achieving *Kt*/*V* ≥ 1.2 and avoiding large interdialytic weight gain [2]. Although the mortality rate of patients with ESKD is improving, it remains higher than that of the general population, with cardiovascular events being the topmost contributor to mortality [3, 4].

Body mass index (BMI) is a strong predictor of mortality in patients undergoing haemodialysis (HD). The relationship between high BMI and better survival has been reported in several HD cohorts and is termed the obesity paradox [5, 6, 7]. In addition to baseline BMI, the pattern of weight change favours weight gain for better survival in many, but not all, studies, whereas weight loss is consistently associated with an increased risk of death in the HD population [7, 8, 9]. In the Asian population, this paradox is less consistent, with increased mortality associated with a higher BMI in some cohort studies [10, 11]. A nationwide Korean HD registry survey showed an early survival advantage associated with obesity; however, its effect waned over time with a surge in mortality among young obese individuals [12]. In addition, the obesity paradox has been challenged by the possibility of reverse causation, indicating that healthy individuals do not experience illness‐related weight loss and show better survival [13]. Thus, the health benefits of targeting higher body weight have been questioned.

The current BMI classification is primarily derived from large epidemiological studies focusing on all‐cause mortality, with less emphasis on obesity‐related morbidities such as cardiovascular events [14]. To our knowledge, previous studies evaluating all‐cause and cardiovascular mortality according to baseline BMI and body weight changes in patients undergoing HD were limited by the exclusion of cardiovascular morbidities [5, 6, 7, 8, 9, 10, 11, 12, 15, 16, 17].

Accurate weight determination is challenging in patients undergoing HD because it is difficult to differentiate between volume overload and lean body mass. By definition, dry weight is the lowest tolerated post‐dialysis weight achieved at which there are minimal signs or symptoms of hypovolaemia or hypervolaemia. Therefore, dry weight may be a more accurate and readily measurable determinant of body weight in patients undergoing HD. In this secondary analysis of a prospective cohort study, we examined dry weight changes in a cohort of Korean patients undergoing HD and investigated their association with patient survival and major adverse cardiovascular events (MACEs). We hypothesized that dry weight changes, including both weight gain and loss, would be associated with increased mortality and cardiovascular events.

## 2 Methods

### 2.1 Study Population and Design

**FIGURE 1:**
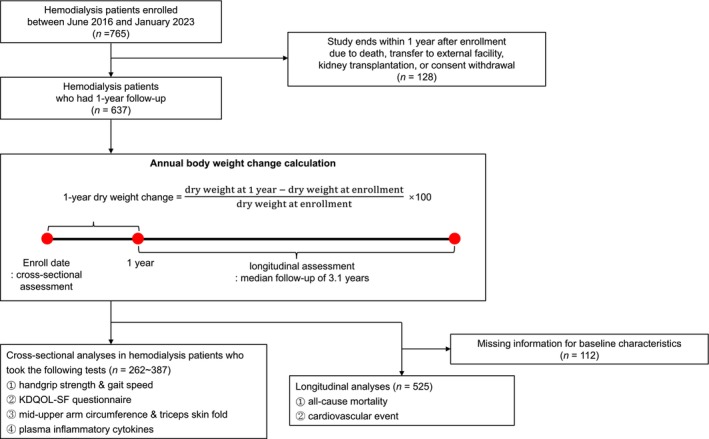
Study population and design.

This study used data obtained from the K‐cohort. K‐cohort is a multicentre, observational, prospective cohort comprising patients undergoing thrice‐weekly HD (CRIS No. KCT0003281). Since this study utilized data from a pre‐established cohort, a priori sample size calculation was not applicable. We included patients older than 18 years receiving thrice‐weekly HD and excluded those who were pregnant or were expected to have a life expectancy of less than 6 months. Of the 765 patients enrolled from seven dialysis centres in the Republic of Korea between June 2016 and January 2023, 525 patients with follow‐up data for at least 1 year were included for cross‐sectional and longitudinal analyses (Figure 1).

Dry weight change was calculated as the difference between dry weight at enrolment and at 1 year, divided by dry weight at enrolment. Patients were stratified into five groups according to annual dry weight change (Figure S1): stable (−2.0% to 1.9%), mild (2.0% to 6.9%) and moderate (≥ 7.0%) dry weight gain and mild (−5.0% to −2.1%) and moderate (< −5.0%) dry weight loss. Patients were also classified as underweight (< 18.5 kg/m^2^), normal (18.5–22.9 kg/m^2^), overweight (23.0–24.9 kg/m^2^) and obese (≥ 25 kg/m^2^) based on baseline BMI according to the World Health Organization Asia‐Pacific classification of weight status [18].

### 2.2 Data Collection

Demographic and clinical data, including age, sex, dry weight, height, predialysis blood pressure, diabetes mellitus status and medical history of coronary artery disease and cerebral infarction, were collected at the time of enrolment through review of medical records. Dialysis‐related parameters, such as vascular access type, dialysis modality, blood flow rate, dialysis duration, convection volume, single‐pool *Kt*/*V* (sp*Kt*/*V*) and dialysis vintage, were also ascertained at enrolment. Comorbidities were assessed to calculate the age‐unadjusted Charlson comorbidity index [19]. Dry weight was defined as the clinician's assessment of the lowest attainable post‐dialysis body weight without oedema or intradialytic complications such as hypotension or cramping. BMI was calculated as dry weight (kilogramme) divided by the square of height (square metre). Data on dry weight, vascular access, blood flow rate, dialysis duration and sp*Kt*/*V* were collected at baseline and 1 year after enrolment. sp*Kt*/*V*, where *K* represents urea clearance by the dialyser, *t* represents treatment time and *V* represents the volume of urea distribution, was calculated using the conventional two‐point urea modelling method. The following dialysers were used: FX8, FX10, Polyflux 14L and Polyflux 17L for low‐flux HD and FX60, FX80, FX600, FX800 and Polyflux 170H for high‐flux HD or haemodiafiltration (HDF). As part of routine clinical care, laboratory values of haemoglobin, albumin, sodium, potassium, calcium, phosphate and C‐reactive protein were measured at each centre using pre‐dialysis blood samples collected during a midweek HD session. All laboratory parameters except C‐reactive protein were measured every 6 months, and the 1‐year average values were used for analysis.

### 2.3 Questionnaire for Quality of Life

At enrolment, patients were asked to complete the Korean version of the Kidney Disease Quality of Life‐Short Form (KDQOL‐SF) questionnaire, a self‐reported survey designed to assess quality of life across physical, mental and kidney disease–specific domains. The scales for the physical component summary (PCS) and mental component summary (MCS) were calculated from eight subscales [20]: physical functioning, role‐physical, bodily pain, general health, vitality, social functioning, role‐emotional and mental health. The kidney disease component summary (KDCS) scale was computed as the average of seven subscales as previously described [21]: symptom/problem list, effects of kidney disease, burden of kidney disease, cognitive function, quality of social interaction, sleep and social support. Because of weighting, PCS scales ranged from 1.7 to 76.3, and MCS scales ranged from 1.3 to 80.7. KDCS scales ranged from 0 to 100. For all scales, higher scores indicate better quality of life and less impairment. After excluding patients who refused to complete the survey, the analysis was conducted on a subset of patients who had completed the questionnaire for each scale at baseline and at 1 year.

### 2.4 Measurements of Gait Speed and Handgrip Strength

As previously described [22], gait speed was assessed within 1 month of enrolment, following the completion of a dialysis session on a treatment day with a short interdialytic interval. Gait speed was measured by manually timing participants as they walked a 4‐m course at their usual pace. The average speed from three trials was used for analysis. Handgrip strength was measured using a Jamar hand dynamometer (Sammons Preston Inc., Bolingbrook, IL, USA) on the non‐access side of the hand during a dialysis session. The highest value among three measurements was recorded. In accordance with the criteria established in 2019 by the Asian Working Group for Sarcopenia [23], a slow gait speed was defined as < 1.0 m/s, and weak handgrip strength was defined as < 26 kg for men and < 18 kg for women.

### 2.5 Mid‐Arm Measurements

Mid‐upper arm circumference (MUAC) and triceps skin‐fold (TSF) thickness were measured on the non‐access arm using standard techniques [24]. MUAC was assessed at the midpoint between the acromion and olecranon, with the elbow fully extended. TSF was measured at the same point along the posterior aspect of the arm. The mid‐arm muscle circumference (MAMC) was calculated using the following formula: MAMC (centimetre) = MUAC − π × TSF thickness (centimetre).

### 2.6 Measurement of Plasma Inflammatory Cytokines

Blood samples were collected at enrolment in ethylenediaminetetraacetic acid–treated tubes and centrifuged at 1000*g* for 15 min. The supernatants were stored at −80°C until analysis. For participants enrolled before July 2019, stored plasma samples were used to simultaneously quantify multiple inflammatory markers, including interleukin (IL)‐6, IL‐18, tumour necrosis factor‐α, monocyte chemoattractant protein‐1, a proliferation‐inducing ligand and B cell–activating factor, using a multiplex enzyme‐linked immunosorbent assay with Magnetic Luminex Screening Assay kits (R&D Systems Inc., Minneapolis, MN, USA).

### 2.7 Study Outcomes

The study endpoints included death from any cause and a composite of MACE, defined as myocardial infarction, unstable angina, ischaemic stroke and peripheral artery disease requiring revascularization. For sensitivity analyses, MACE plus stable angina treated with percutaneous coronary intervention (PCI) or coronary artery bypass graft (CABG) were also examined. All deaths and cardiovascular events were retrieved and carefully reviewed. Patients were censored if they transferred to other facilities (*n* = 57), underwent kidney transplantation (*n* = 30), withdrew consent (*n* = 24), or switched to peritoneal dialysis (*n* = 4), whichever occurred first.

### 2.8 Statistical Analysis

Data are presented as mean ± standard deviation or median (interquartile range [IQR]) for continuous variables and as absolute number (percentage) for categorical variables. Continuous variables were first tested for normality using the Shapiro–Wilk test and evaluated for intergroup comparisons using one‐way analysis of variance or the Kruskal–Wallis test, as appropriate. Categorical variables were analysed using the *χ* ^2^ test or Fisher's exact test.

For cross‐sectional analysis, patients with at least 1 year of follow‐up were stratified into three groups based on handgrip strength and gait speed: (1) normal handgrip strength and gait speed, (2) weak handgrip strength or slow gait speed and (3) weak handgrip strength and slow gait speed. Further, patients were divided into tertiles according to their PCS, MCS and KDCS scores derived from the KDQOL‐SF questionnaire. The distribution patterns of annual dry weight changes across these tertiles were visualized using kernel density estimation. Annual dry weight changes were then classified as stable (−2.0% to 1.9%), weight gain (≥ 2.0%) and weight loss (≤ −2.1%). Comparisons were made between these categories and the tertiles of handgrip strength and gait speed, PCS, MCS and KDCS scores. Additionally, anthropometric parameters and circulating inflammatory cytokine levels were compared between weight change groups.

In the longitudinal analysis, patients were followed for up to 7 years after the 1‐year observation period. Kaplan–Meier analyses were performed using log‐rank tests to compare the cumulative incidence of study outcomes among the dry weight change groups. Survival time was defined as the interval between the end of the 1‐year visit and the onset of the study outcomes or May 2024, whichever came first.

Cause‐specific hazards models were constructed to determine the independent association between baseline BMI or dry weight change group and study outcomes, including all‐cause mortality or MACE (or MACE with PCI or CABG). Proportional hazards assumption was assessed using the cox.zph function (Table S1). The following variables considered clinically significant for the outcomes were included in the adjusted models: age, sex, diabetes mellitus, haemoglobin, albumin, sp*Kt*/*V*, HD modality (conventional HD versus HDF), dialysis blood flow and dialysis vintage. The average values of haemoglobin, albumin and sp*Kt*/*V*, measured at baseline, 6‐month and 12‐month visits, were incorporated into multivariable models. Regarding MACE or MACE plus stable angina treated with PCI or CABG, subdistribution hazard models, in which death that occurred before the events was considered as a competing event, were also constructed using the method of Fine and Grey [25] with the same covariables that were adjusted in the cause‐specific hazards models. Subgroup analysis was performed to examine the differential effects of dry weight changes on all‐cause mortality according to baseline BMI, average albumin levels and age. The underweight group was excluded from subgroup analysis because of a relatively small number of patients. A *p* value < 0.05 was considered to be statistically significant. All statistical analyses were performed using R software Version 4.4.1.

## 3 Results

### 3.1 Clinical Features Associated With Body Weight Changes

**FIGURE 2:**
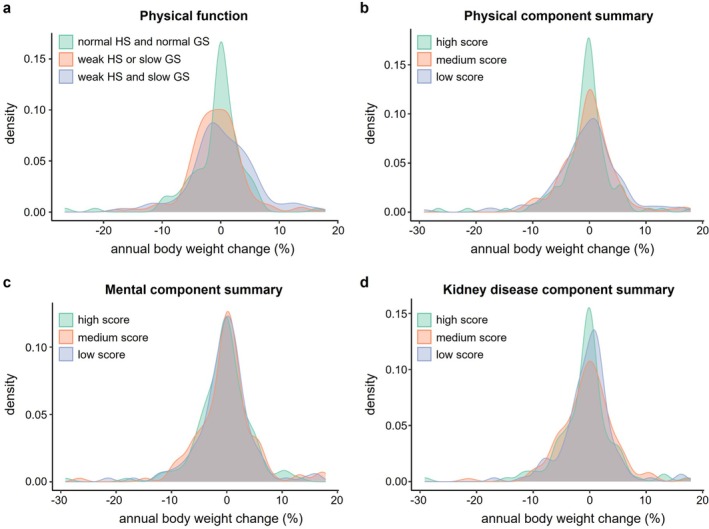
Distribution of annual body weight change according to physical function and three components of health‐related quality of life. (a) Kernel density plot showing the distribution of annual body weight change across three groups stratified by handgrip strength (HS) and gait speed (GS): normal HS and normal GS, weak HS or slow GS and weak HS and slow GS. According to Asian Working Group for Sarcopenia 2019 criteria [23], weak HS was defined as < 28.0 kg for men and < 18.0 kg for women, while slow GS was defined as < 1.0 m/s. (b–d) Kernel density plot showing the distribution of annual body weight change according to tertiles of physical (b), mental (c) and kidney disease (d) component summary scores derived from the Kidney Disease Quality of Life, Short Form questionnaire.

We conducted a cross‐sectional analysis to identify characteristics associated with body weight changes among patients undergoing HD. Among those who completed both handgrip strength and 4‐m gait speed assessments, individuals with reduced handgrip strength and/or slower gait speed exhibited a wider distribution of weight changes in both directions than those with preserved physical function (Figure 2a and Table S2). In addition, in patients who completed the KDQOL‐SF questionnaire, lower PCS scores were significantly associated with a higher proportion of weight gain (≥ 2%) or loss (< −2%) (Figure 2b). In contrast, no notable differences in weight change patterns were observed across different levels of MCS and KDCS scores (Figure 2c,d). Regarding MAMC and TSF (surrogates for muscle and subcutaneous fat mass), as well as circulating inflammatory cytokine levels, no significant differences were found among the stable, weight gain, or weight loss groups (Table S2).

### 3.2 Baseline Characteristics

To investigate the long‐term clinical impact of body weight changes, longitudinal analyses were performed in 525 individuals. The baseline characteristics of each group are shown in Table 1. The mean age of the patients was 59.9 ± 12.8 years and 327 (62.3%) of the patients were male. At enrolment, the mean BMI was 23.2 ± 4.1 kg/m^2^, and the mean dry weight was 62.6 ± 12.9 kg with no significant differences among groups. The direction of dry weight change from ‘moderate weight gain’ to ‘stable’, then to ‘moderate weight loss’ was associated with higher age and longer dialysis vintage, as well as an inverted U–shaped pattern in HD blood flow, with the stable group showing the highest value. Although the proportion of patients on HDF in the stable group was higher than that in the weight gain or loss groups, no significant differences were observed across the groups. Notably, only 11 of 144 patients on HDF achieved a high‐volume convection volume (at least 46 and 23 L per session in predilution and postdilution mode, respectively). As catheter use at baseline transitioned to fistula or graft access after 1 year, the type of vascular access became similar among the groups. In addition, no significant differences were observed in comorbidities, haemoglobin, electrolyte and C‐reactive protein levels, HD duration per session and sp*Kt*/*V*. According to baseline BMI, overweight patients showed a significantly lower risk of all‐cause mortality than normal weight patients (adjusted hazard ratio [aHR], 0.53; 95% confidence interval [CI], 0.29–0.97; *p* value = 0.04), but not obese patients (Figure S2a). Moreover, overweight patients showed a trend towards a lower risk of MACE compared to normal weight patients (Figure S2b).

**TABLE 1:** Baseline characteristics of the study population according to annual dry weight change.

	Moderate weight gain (*n* = 20)	Mild weight gain (*n* = 92)	Stable (*n* = 245)	Mild weight loss (*n* = 91)	Moderate weight loss (*n* = 77)	*p*
Age (year)	55.5 ± 14.5	57.7 ± 12.5	59.3 ± 12.5	62.4 ± 11.6	63.0 ± 14.0	0.003
Male sex	8 (40.0)	59 (64.1)	157 (64.1)	59 (64.8)	44 (57.1)	0.21
BMI (kg/m^2^)	23.2 ± 6.5	22.9 ± 3.4	23.3 ± 4.1	23.0 ± 4.1	23.7 ± 4.2	0.62
Dry weight at baseline (kg)	59.5 ± 16.8	62.0 ± 11.0	63.2 ± 12.8	62.7 ± 13.6	62.0 ± 13.6	0.37
Dry weight after 1 year (kg)	66.7 ± 18.4	64.4 ± 11.4	63.3 ± 12.9	60.6 ± 13.1	56.5 ± 12.7	< 0.001
Height (cm)	160.1 ± 9.4	164.2 ± 8.0	164.5 ± 9.0	164.8 ± 8.2	161.5 ± 9.0	0.04
Blood pressure before dialysis (mmHg)						
Systolic^a^	142.4 ± 25.1	143.2 ± 18.9	146.2 ± 22.2	146.5 ± 21.0	146.1 ± 18.5	0.78
Diastolic^a^	69.1 ± 15.4	73.0 ± 12.3	71.3 ± 13.6	70.3 ± 11.6	70.8 ± 14.7	0.53
Comorbidity						
Diabetes mellitus	14 (70.0)	48 (52.2)	138 (56.3)	51 (56.0)	43 (55.8)	0.71
Coronary artery disease	5 (25.0)	22 (23.9)	47 (19.2)	16 (17.6)	18 (23.4)	0.70
Cerebral infarction	3 (15.0)	21 (22.8)	31 (12.7)	14 (15.4)	7 (9.1)	0.11
Charlson comorbidity index^a^	4.2 ± 1.2	4.2 ± 1.7^b^	4.0 ± 1.6^a^	4.0 ± 1.5	4.1 ± 1.8	0.90
Laboratory values						
Haemoglobin (g/dL)^b^	10.8 ± 0.9	10.7 ± 0.8	10.7 ± 0.9	10.6 ± 0.8	10.4 ± 0.8	0.21
Albumin (g/dL)^b^	3.9 ± 0.3	3.9 ± 0.3	4.0 ± 0.3	3.9 ± 0.3	3.8 ± 0.3	0.003
Na (mmol/L)^b^	134.8 ± 11.2	137.4 ± 3.2	137.5 ± 3.9	136.9 ± 3.4	137.2 ± 2.9	0.32
K (mmol/L)^b^	4.8 ± 0.7	4.9 ± 0.6	4.9 ± 0.6	4.9 ± 0.6	4.8 ± 0.5	0.39
Ca (mg/dL)^b^	8.5 ± 0.7	8.7 ± 0.6	8.7 ± 0.7	8.7 ± 0.6	8.6 ± 0.7	0.69
P (mg/dL)^b^	5.7 ± 1.4	4.9 ± 0.9	5.0 ± 1.0	4.9 ± 1.1	4.9 ± 1.1	0.26
C‐reactive protein (mg/L)^a^	1.1 (0.5**–**4.6)	1.7 (0.6**–**3.8)	1.2 (0.4**–**3.1)	2.0 (0.6**–**4.2)	1.6 (0.6**–**5.3)	0.15
Vascular access at baseline						0.02
Fistula	14 (70.0)	82 (89.1)	210 (85.7)	80 (87.9)	63 (81.8)	
Graft	2 (10.0)	5 (5.4)	24 (9.8)	9 (9.9)	12 (15.6)	
Catheter	4 (20.0)	5 (5.4)a	11 (4.5)	2 (2.2)	2 (2.6)	
Vascular access after 1 year						0.38
Fistula	17 (85.0)	84 (91.3)	216 (88.2)	81 (89.0)	62 (80.5)	
Graft	3 (15.0)	6 (6.5)	24 (9.8)	10 (11.0)	13 (16.9)	
Catheter	0 (0.0)	2 (2.2)	5 (2.0)	0 (0.0)	2 (2.6)	
Haemodialysis modality						0.34
Haemodialysis	16 (80.0)	69 (75.0)	168 (68.6)	67 (73.6)	61 (79.2)	
Low flux	3 (15.0)	5 (5.4)	9 (3.7)	4 (4.4)	4 (5.2)	
High flux	13 (65.0)	64 (69.6)	159 (64.9)	63 (69.2)	57 (74.0)	
Haemodiafiltration	4 (20.0)	23 (25.0)	77 (31.4)	24 (26.4)	16 (20.8)	
Predilution	2 (10.0)	3 (3.3)	17 (6.9)	6 (6.6)	5 (6.5)	
Postdilution	2 (10.0)	20 (21.7)	60 (24.5)	18 (19.8)	11 (14.3)	
Dialysis parameters						
Blood flow (mL/min)^d^	250 (230**–**275)	268 (255**–**290)	280 (260**–**300)	270 (250**–**285)	260 (250**–**280)	< 0.001
Duration (hour/session)^d^	4.0 ± 0.1	3.8 ± 0.3	3.8 ± 0.3	3.9 ± 0.2	3.8 ± 0.3	0.06
Convection volume (L/session)^c^	18.5 (16.0**–**21.1)	18.0 (16.5**–**18.0)	18.0 (17.0**–**20.0)	18.0 (17.0**–**20.3)	18.6 (17.0**–**23.3)	0.45
Single‐pool *Kt*/*V* ^d^	1.6 ± 0.3	1.6 ± 0.2	1.6 ± 0.3	1.6 ± 0.3	1.6 ± 0.3	0.90
Dialysis vintage (year)	0.63 (0.32**–**2.69)	2.52 (0.95**–**7.06)	2.89 (1.05**–**7.05)	3.27 (1.41**–**8.69)	3.93 (2.20**–**8.49)	0.006

*Note:* Data are expressed as mean ± standard deviation, median (interquartile range) or number (%).

^a^ Missing value (*n* = 1 for blood pressure, *n* = 2 for Charlson comorbidity index and *n* = 47 for C‐reactive protein).

^b^ Values measured at enrolment and at 6 and 12 months after enrolment were averaged.

^c^ Values were obtained from participants who underwent haemodiafiltration. For the predilution mode, half of the convection volume was incorporated, whereas the original convection volume was used for the postdilution mode.

^d^ Values measured at enrolment and at 12 months after enrolment were averaged.

### 3.3 All‐Cause Mortality According to Annual Dry Weight Change

**FIGURE 3:**
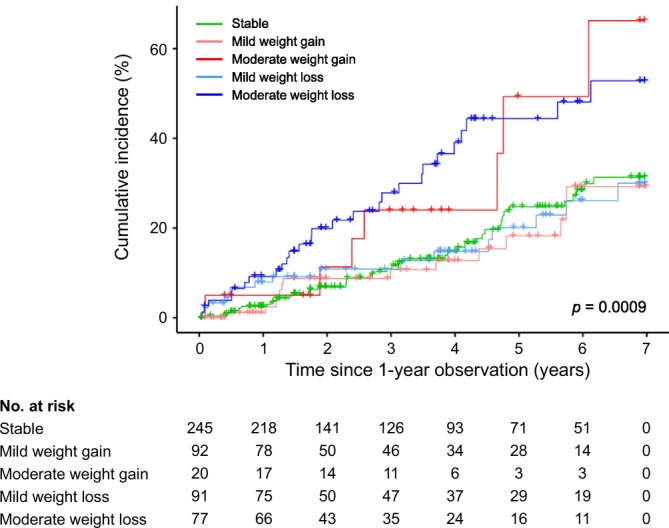
Kaplan–Meier curves for all‐cause death according to annual dry weight change.

During a median follow‐up of 3.1 years (IQR, 1.5–5.6 years), 105 (20.0%) patients died. The Kaplan–Meier curves for all‐cause mortality significantly differed among the five groups categorized by annual dry weight change (Figure 3). Notably, the incidence of all‐cause death was higher in patients with moderate weight gain (35.0%) or moderate weight loss (35.1%) than in the other groups (15.2%–17.6%) (Table 2). In the univariable model, the hazard ratios for all‐cause mortality in the moderate weight gain and moderate weight loss groups, compared with those in the stable group, were 2.27 (95% CI, 1.02–5.07; *p* = 0.04) and 2.44 (95% CI, 1.50–3.96; *p* = 0.0003), respectively (Table 2). Mild weight gain or loss groups were not associated with an increased risk of all‐cause mortality compared to the stable group. After adjustment for age, sex and diabetes mellitus as well as laboratory and dialysis‐related variables, the risk for all‐cause mortality remained significant in the moderate weight loss group (aHR, 2.35; 95% CI, 1.07–2.95; *p* = 0.03) and was marginally significant in the moderate weight gain group (aHR, 1.81; 95% CI, 0.96–5.13; *p* = 0.06). Among the covariables, older age, diabetes mellitus, lower albumin levels and longer dialysis vintage were independently associated with increased mortality (Table S3a).

**TABLE 2:** Hazard ratios for all‐cause mortality according to annual dry weight change.

All‐cause mortality	Death/patients (%)	Death/1000 person‐year	Univariable analysis		Multivariable analysis	
			HR (95% CI)	*p*	HR (95% CI)	*p*
Stable	41/245 (16.7)	47.6	Reference		Reference	
Mild weight gain	14/92 (15.2)	44.8	0.94 (0.51**–**1.73)	0.84	0.92 (0.50**–**1.70)	0.82
Moderate weight gain	7/20 (35.0)	105.4	2.27 (1.02**–**5.07)	0.04	2.22 (0.96**–**5.13)	0.06
Mild weight loss	16/91 (17.6)	51.2	1.06 (0.60**–**1.90)	0.83	0.73 (0.40**–**1.32)	0.30
Moderate weight loss	27/77 (35.1)	112.9	2.44 (1.50**–**3.96)	0.0003	1.78 (1.07–2.95)	0.03

Abbreviations: CI, confidence interval; HR, hazard ratio.

### 3.4 Cardiovascular Events According to Annual Dry Weight Change

**FIGURE 4:**
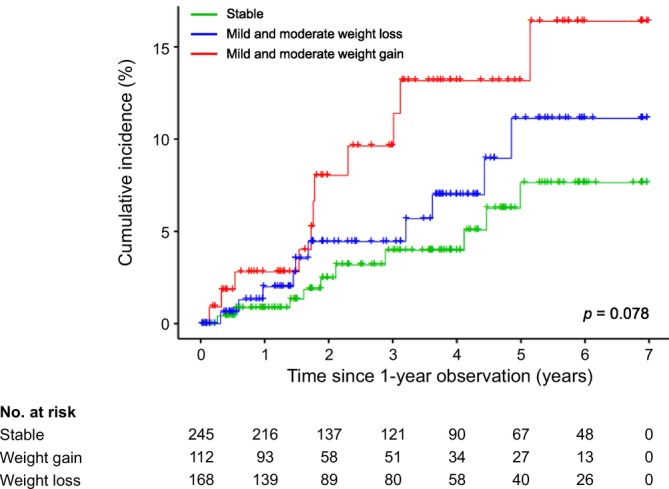
Kaplan–Meier curves for major adverse cardiovascular events according to annual dry weight change.

MACE occurred in 31 patients (5.9%) during the study period. Owing to the relatively low incidence of MACE, we combined the mild and moderate dry weight change groups for analysis. MACE occurrence was highest in the weight gain group, followed by the weight loss group and the stable group (Table 3). Kaplan–Meier curves showed a similar cumulative incidence rate of MACE among these groups (Figure 4). Multivariable Cox regression analysis revealed that the weight gain group had a significantly higher risk of MACE than the stable group (aHR, 2.98; 95% CI, 1.24–7.16; *p* = 0.01) (Table S4a). By contrast, the weight loss group did not demonstrate a significant increase in MACE risk relative to the stable group. Because a larger number of deaths relative to MACE may influence the risk, competing risk analyses were performed. The results remain consistent after deaths were treated as a competing event without censoring, reconfirming the association of weight gain with the development of MACE (aHR, 3.02; 95% CI, 1.32–6.88; *p* = 0.009) (Table 3a). Furthermore, similar findings were observed for a broader cardiovascular outcome, MACE plus stable angina requiring revascularization, using both standard Cox and competing risk hazards models (Tables 3b and S4b). Similar to the analyses for all‐cause mortality, older age and diabetes mellitus were associated with MACE occurrence among the covariables (Table S3b).

**TABLE 3:** Subdistribution hazard ratios for MACE according to annual dry weight change.

(a) Hazard ratio for MACE						
MACE	Events/patients (%)	Events/1000 person‐year	Univariable analysis		Multivariable analysis	
			HR (95% CI)	*p*	HR (95% CI)	*p*
Stable	10/245 (4.1)	11.9	Reference		Reference	
Mild and moderate weight gain	11/112 (9.8)	31.6	2.60 (1.11–6.08)	0.03	3.02 (1.32–6.88)	0.009
Mild and moderate weight loss	10/168 (6.0)	18.9	1.49 (0.62**–**3.57)	0.37	1.33 (0.53–3.33)	0.54

(b) Hazard ratio for MACE plus stable angina with PCI or CABG						
MACE plus stable angina with PCI or CABG	Events/patients (%)	Events/1000 person‐year	Univariable analysis		Multivariable analysis	
			HR (95% CI)	*p*	HR (95% CI)	*p*
Stable	21/245 (8.6)	26.3	Reference		Reference	
Mild and moderate weight gain	17/112 (15.2)	52.1	1.94 (1.03–3.66)	0.04	2.09 (1.09–4.01)	0.03
Mild and moderate weight loss	20/168 (11.9)	39.7	1.43 (0.78**–**2.62)	0.25	1.25 (0.65–2.42)	0.51

*Note:* HRs were estimated using all‐cause death as a competing event.

Abbreviations: CABG, coronary artery bypass graft; CI, confidence interval; HR, hazard ratio; MACE, major adverse cardiovascular events; PCI, percutaneous coronary intervention.

### 3.5 Subgroup Analyses Based on Baseline BMI, Average Albumin Levels and Age

**FIGURE 5:**
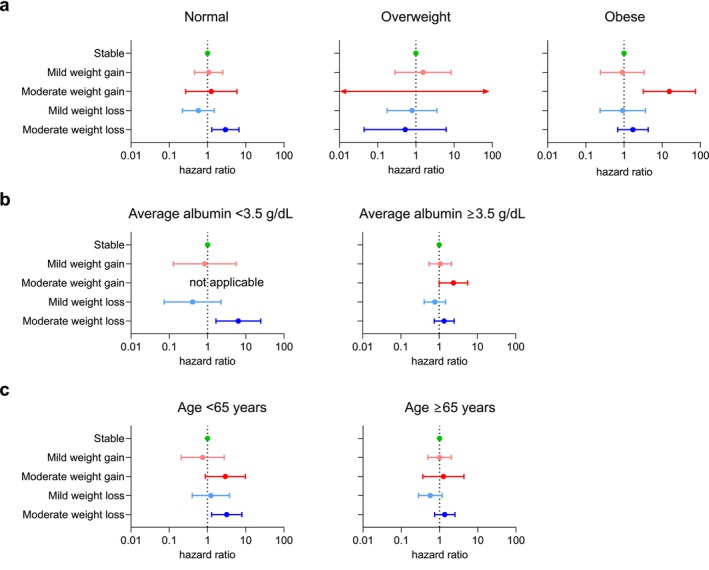
Forest plot presenting hazard ratios for all‐cause mortality according to annual dry weight change. In subgroups stratified by baseline body mass index (a), average serum albumin levels (b) and age (c), the hazard ratios and confidence intervals were estimated from Cox proportional‐hazards models with adjustment for age, sex, diabetes mellitus, average haemoglobin, average serum albumin, single‐pool *Kt*/*V*, haemodialysis modality (conventional haemodialysis versus haemodiafiltration) and dialysis vintage. The closed circle represents the hazard ratio, with the horizontal line indicating the 95% confidence interval.

To assess whether the association between annual dry weight change and all‐cause mortality differed according to patient characteristics, we performed subgroup analyses stratified by baseline BMI, average serum albumin levels and age. When stratified by baseline BMI, the impact of annual dry weight changes on all‐cause mortality differed across BMI categories (Figure 5a). Among patients with initially normal BMI, those who experienced moderate weight loss (Table S5), but not moderate weight gain, had a higher risk of all‐cause mortality (aHR, 2.94; 95% CI, 1.29–6.67; *p* = 0.01). Conversely, among patients who were initially obese, those who experienced moderate weight gain, but not moderate weight loss, had a higher risk of all‐cause mortality (aHR, 15.43; 95% CI, 3.20–74.50; *p* = 0.0007). These findings suggest that weight changes towards both extremes were associated with lower survival among patients undergoing HD.

A similar pattern was observed when participants were stratified by serum albumin level (Figure 5b). Among patients with hypoalbuminemia (albumin < 3.5 g/dL, indicating malnutrition), moderate weight loss (Table S6) was associated with increased mortality (aHR, 6.38; 95% CI, 1.66–24.57; *p* = 0.007). In contrast, among patients with serum albumin levels ≥ 3.5 g/dL (indicative of good nutritional status), moderate weight gain was linked to a higher risk of all‐cause mortality (aHR 2.37; 95% CI, 1.01–5.60; *p* value = 0.049).

Finally, when stratified by age, the association between dry weight change and mortality was only evident among younger patients. In those aged < 65 years, moderate weight loss was associated with increased mortality compared with stable weight (aHR, 3.20; 95% CI, 1.29–7.96; *p* = 0.01), while moderate weight gain showed a non‐significant trend towards higher mortality (aHR, 2.93; 95% CI, 0.88–9.75; *p* = 0.08) (Figure 5c and Table S7). However, in patients aged ≥ 65 years, dry weight changes in either direction were not associated with all‐cause mortality, suggesting that the detrimental effects of weight instability may be more pronounced in younger individuals.

## 4 Discussion

In this multicentre study conducted in South Korea, we examined the association between annual dry weight change and clinical outcomes, including all‐cause mortality and cardiovascular events, in patients undergoing HD. Our findings underscore the potential risks associated with both weight gain and weight loss in this population. Notably, physically frail individuals were particularly vulnerable to body weight fluctuations, potentially due to decreased physical activity or underlying catabolic conditions. These results align with previous observational studies, which have reported lower survival rates among both incident and prevalent HD patients who experienced weight loss [6, 7, 8, 9, 15, 16, 17, 26]. Additionally, most of these studies suggested a survival benefit associated with weight gain in HD patients, particularly in Western populations. Such findings may have contributed to a more permissive clinical attitude towards weight management in obese patients receiving HD.

However, the associations between weight gain and improved survival were not consistently significant in unadjusted analyses in some studies [6, 16]. Notably, a French cohort study even reported an increased risk of mortality among older HD patients who gained weight [17]. Furthermore, several observational studies conducted in Asian populations have shown that obese patients undergoing HD or peritoneal dialysis had higher mortality rates [10, 11, 27]. Taken together with our findings, these results suggest that nutritional strategies aimed at increasing body weight should be implemented with caution. Given that patients on HD may be candidates for kidney transplantation, maintaining a healthy body weight is particularly important to facilitate surgical access and optimize post‐transplant outcomes.

An important factor to consider when evaluating the health effects of body weight change is the duration of exposure following the weight change. Weight loss tends to have a relatively rapid negative impact on health, often manifesting within a few years, whereas the adverse effects of weight gain on health outcomes develop more gradually [28, 29]. Previous studies had median or mean follow‐up periods of approximately 2 years [6, 7, 8, 9, 16], which may be insufficient to observe the long‐term consequences of weight gain. By following the patients for up to 7 years, our study calls into question the health benefits of weight gain in HD patients. The Kaplan–Meier curve (Figure 3) visually demonstrates that the difference in cumulative incidence of all‐cause mortality between the moderate weight gain and stable weight groups begins to emerge after 2 years of follow‐up. In addition, the investigation of cardiovascular outcomes revealed that an increased risk of MACE in the weight gain group was observed before its effect on mortality became apparent, suggesting a sequential causal relationship in which cardiovascular events may precede and contribute to death.

Weight loss is an ominous sign that warrants close attention. In our subgroup analyses, the association between weight loss and increased mortality was significant only among individuals with a normal BMI or lower serum albumin levels, suggesting a role of protein‐energy wasting in this relationship. Particularly, the catabolic state commonly accompanied by inflammation and oxidative stress may contribute to decreased survival in this subgroup [30, 31, 32]. Conversely, the adverse effect of weight gain was primarily observed in individuals with obesity or normal albumin levels. In these patients, as in the general population, excess caloric intake may exacerbate metabolic comorbidities and accelerate atherosclerosis, thereby increasing the risk of cardiovascular events and mortality.

Age was a significant risk factor for mortality in our cohort. Specifically, the incidence of all‐cause death was 72 of 206 (35.0%) among patients aged ≥ 65 years, compared with 33 of 319 (10.3%) among those aged < 65 years. Our study population was relatively young, considering that the average age at the initiation of kidney replacement therapy is over 65 years [33, 34]. Notably, body weight changes did not significantly affect all‐cause mortality among older patients. This finding is consistent with recent findings that both the negative impact of lower BMI and the protective effect of higher BMI on mortality become attenuated with increasing age in Korean HD patients [35]. We speculate that the influence of body weight change on death may have been relatively diminished due to the overriding influence of age.

Similar to our definition, previous studies defined stable dry weight as < ±1%–2% [6, 8, 9, 15, 16]. Generally, > 5% weight reduction was recommended to achieve health benefits in overweight and obese people [36, 37]. Although suboptimal, even a modest weight loss of 3%–5% results in clinically meaningful health advantages. The clinical context of weight change in this study (regarded as a natural change) differs from those of the guidelines aimed at reducing obesity complications in the general population (intentional for health promotion). Despite these contextual differences, our results suggest that similar magnitudes of weight change, whether towards more obese or underweight, adversely affect the health outcomes in the HD population.

Compared to the CONVINCE trial with a median follow‐up of 30 months [38], the mortality rate in our study (20.0%) was similar to that observed in the HD arm (21.9%) and higher than that in the HDF arm (17.3%). This comparison implies that HDF implementation would likely have had little impact on survival in our cohort. Overall, only 144 patients (27.4%) received HDF and the average convection volume in each group ranged from 18.0 to 18.6 L/session, which does not satisfy the recommended convection volume in a recent consensus statement [39]. Baseline dialysis parameters suggested that securing adequate blood flow was a major barrier to delivering high‐volume HDF in our study, particularly for participants who experienced weight changes.

Our study has some limitations that should be noted. First, this study was observational and there were unbalanced baseline variables such as age, dialysis blood flow and dialysis vintage among the weight change groups. To account for potential confounding factors, we adjusted for patient and dialysis factors that might influence clinical outcomes. Second, the study lacked information on the cause of death, intentionality of weight change, smoking habit and weight changes according to body weight composition (muscle or fat). Third, the moderate sample size of our cohort may have limited the statistical significance of the association between moderate weight gain and all‐cause mortality, which was marginally significant (*p* = 0.06). Finally, the study population included only Koreans, unlike previous studies that predominantly included White and Black populations [6, 7, 9, 15, 16, 17]. Despite being mono‐ethnic, our study offers valuable real‐world insights into Asian populations, which have been studied less frequently in this context. It is also worth mentioning that by using dry weight, we aimed to minimize the potential bias from excess fluid in our assessment of actual body weight changes.

In conclusion, this study demonstrated that body weight changes to become more obese or underweight were associated with an increased risk of all‐cause mortality, with weight gain increasing the risk of cardiovascular events. Our study emphasizes the importance of maintaining a healthy body weight to improve the overall survival of patients undergoing HD. As dialysis technology continues to advance with the broader adoption of high‐volume HDF, the life expectancy in this population is expected to increase. Consequently, long‐term adverse effects of weight gain are likely to become increasingly important in the management of patients undergoing HD.

## Ethics Statement

The authors certify that they comply with the ethical guidelines for publishing in the *Journal of Cachexia, Sarcopenia and Muscle* [40]. The study was conducted in accordance with the Declaration of Helsinki, and the study protocol was approved by the institutional review boards of all participating centres (KHNMC IRB No. 2016‐04‐039). Written informed consent was obtained from all participants before enrolment.

## Conflicts of Interest

The authors declare no conflicts of interest.

## Supporting information


**Table S1:** Assessment of the proportional hazard assumption.
**Table S2:** Physical function, KDQOL‐SF scores, anthropometric measurements and inflammatory cytokine levels according to annual dry weight changes.
**Table S3:** Hazard ratios of covariables.
**Table S4:** Cause‐specific hazard ratios for cardiovascular events according to annual dry weight change.
**Table S5:** BMI at baseline and after 1 year stratified by baseline BMI.
**Table S6:** BMI at baseline and after 1 year stratified by average serum albumin level.
**Table S7:** BMI at baseline and after 1 year stratified by age.
**Figure S1:** Distribution of annual dry weight change in the study population.
**Figure S2:** Forest plot showing hazard ratios for all‐cause mortality and MACE according to baseline body mass index.
