# Supplementary material for: Impact of Annual Dry Weight Changes on Mortality and Cardiovascular Outcomes in Patients Undergoing Haemodialysis
Source: J Cachexia Sarcopenia Muscle. 2025 Oct 14;16(5):e70100. doi: 10.1002/jcsm.70100 (PMC12519516; doi:10.1002/jcsm.70100)
Supplement: Supplementary file 1 — Table S1: Assessment of the proportional hazard assumption. Table S2: Physical function, KDQOL‐SF scores, anthropometric measurements and inflammatory cytokine levels according to annual dry weight changes. Table S3: Hazard ratios of covariables. Table S4: Cause‐specific hazard ratios for cardiovascular events according to annual dry weight change. Table S5: BMI at baseline and after 1 year stratified by baseline BMI. Table S6: BMI at baseline and after 1 year stratified by average serum albumin level. Table S7: BMI at baseline and after 1 year stratified by age. Figure S1: Distribution of annual dry weight change in the study population. Figure S2: Forest plot showing hazard ratios for all‐cause mortality and MACE according to baseline body mass index. [file JCSM-16-e70100-s001.docx]

**Journal of Cachexia, Sarcopenia and Muscle**

**Online-Only Supplementary Material**

**Impact of annual dry weight changes on mortality and cardiovascular outcomes in patients undergoing hemodialysis**

Jihoon Park^1,*^, Yoosun Joo^1,2,*^, Yang-Gyun Kim^1,3^, Sang-Ho Lee^1,3^, Ju-Young Moon^1,3^, Hyeon Seok Hwang^3,4^, Jihyun Baek^5^, Dong-Young Lee^6^, Gang Jee Ko^7^, Min-Jeong Lee^8^, Seok Hui Kang^9^, Su Woong Jung^1,3^

^1^Division of Nephrology, Department of Internal Medicine, Kyung Hee University Hospital at Gangdong, Seoul, Republic of Korea
^2^Department of Medicine, Graduate School, Kyung Hee University, Seoul, Republic of Korea
^3^Division of Nephrology, Department of Internal Medicine, College of Medicine, Kyung Hee University, Seoul, Republic of Korea

^4^Division of Nephrology, Department of Internal Medicine, Kyung Hee University Hospital, Kyung Hee University College of Medicine, Seoul, Republic of Korea

^5^Division of Nephrology, Department of Internal Medicine, CHA University Bundang Medical Center, Seongnam, Republic of Korea

^6^Division of Nephrology, Department of Internal Medicine, VHS Medical Center, Seoul, Republic of Korea

^7^Division of Nephrology, Department of Internal Medicine, Korea University College of Medicine, Seoul, Republic of Korea

^8^Division of Nephrology, Department of Internal Medicine, Ajou University Hospital, Suwon, Republic of Korea

^9^Division of Nephrology, Department of Internal Medicine, Yeungnam University College of Medicine, Daegu, Republic of Korea

Correspondence to Su Woong Jung

Division of Nephrology, Department of Internal Medicine, Kyung Hee University Hospital at Gangdong
Email: ha-ppy@daum.net

**Content list**

**Table S1.** Assessment of the proportional hazards assumption

**Table S2.** Physical function, KDQOL-SF scores, anthropometric measurements, and inflammatory cytokine levels according to annual dry weight changes

**Table S3.** Hazard ratios of covariables

**Table S4.** Cause-specific hazard ratios for cardiovascular events according to annual dry weight change

**Table S5.** BMI at baseline and after 1 year stratified by baseline BMI

**Table S6.** BMI at baseline and after 1 year stratified by average serum albumin level

**Table S7.** BMI at baseline and after 1 year stratified by age

**Figure S1.** Distribution of annual dry weight change in the study population

**Figure S2.** Forest plot showing hazard ratios for all-cause mortality and MACE according to baseline body mass index

**Table S1. Assessment of the proportional hazards assumption**

| Variables | *p* value |
| --- | --- |
| Group for 1-year dry weight change | 0.52 |
| Sex | 0.50 |
| Age (per 10 years) | 0.38 |
| Diabetes mellitus | 0.63 |
| Average hemoglobin | 0.88 |
| Average serum albumin | 0.65 |
| Average single-pool Kt/V | 0.55 |
| Hemodialysis modality | 0.08 |
| Dialysis vintage | 0.76 |
| Blood flow (per 10 mL/min) | 0.11 |
| Global | 0.75 |

**Table S2. Physical function, KDQOL-SF scores, anthropometric measurements, and inflammatory cytokine levels according to annual dry weight change**

|  | weight gain | stable | weight loss | *p* value |
| --- | --- | --- | --- | --- |
| **Physical function**^a^ |  |  |  |  |
| Muscle strength and physical performance |  |  |  | 0.02 |
| normal HS and normal GS (*n* = 109) | 24 (22.0) | 57 (52.3) | 28 (25.7) |  |
| weak HS or slow GS (*n* = 127) | 24 (18.9) | 55 (43.3) | 48 (37.8) |  |
| weak HS and slow GS (*n* = 69) | 25 (36.2) | 25 (36.2) | 19 (27.5) |  |
|  |  |  |  |  |
| **KDQOL-SF**^b^ |  |  |  |  |
| Physical component summary |  |  |  | 0.04 |
| high (*n* = 129) | 22 (17.1) | 75 (58.1) | 32 (24.8) |  |
| medium (*n* = 129) | 33 (25.6) | 59 (45.7) | 37 (28.7) |  |
| low (*n* = 129) | 36 (27.9) | 51 (39.5) | 42 (32.6) |  |
| Mental component summary |  |  |  | 0.96 |
| high (*n* = 129) | 32 (24.8) | 58 (45.0) | 39 (30.2) |  |
| medium (*n* = 129) | 30 (23.3) | 63 (48.8) | 36 (27.9) |  |
| low (*n* = 129) | 29 (22.5) | 64 (49.6) | 36 (27.9) |  |
| Kidney disease component summary |  |  |  | 0.36 |
| high (*n* = 116) | 22 (19.0) | 60 (51.7) | 34 (29.3) |  |
| medium (*n* = 116) | 31 (26.7) | 49 (42.2) | 36 (31.0) |  |
| low (*n* = 116) | 23 (19.8) | 63 (54.3) | 30 (25.9) |  |
|  |  |  |  |  |
| **Anthropometric measures** |  |  |  |  |
| Mid-arm muscle circumference (cm) |  |  |  |  |
| Men^c^ | 22.7 (20.4–24.8) | 23.4 (21.3–25.8) | 22.9 (21.7–25.7) | 0.18 |
| Women^d^ | 20.8 (19.7–22.2) | 19.9 (18.4–22.8) | 20.3 (19.1–21.7) | 0.51 |
| Triceps skinfold thickness (cm) |  |  |  |  |
| Men^c^ | 1.4 (1.0–2.3) | 1.0 (1.0–2.0) | 1.1 (1.0–2.0) | 0.64 |
| Women^d^ | 1.5 (1.1–2.2) | 1.7 (1.0–2.4) | 1.6 (1.0–2.1) | 0.94 |
|  |  |  |  |  |
| **Inflammatory cytokines** |  |  |  |  |
| Interleukin-6 (pg/mL)^e^ | 3.1 (2.1–4.6) | 2.8 (1.8–4.3) | 3.5 (2.1–5.0) | 0.19 |
| Interleukin-18 (pg/mL)^e^ | 215.4 (165.6–302.2) | 228.7 (173.1–308.1) | 227.4 (159.8–299.6) | 0.80 |
| Tumor necrosis factor α (pg/mL)^e^ | 10.2 (7.3–13.2) | 10.2 (7.2–13.2) | 10.8 (6.7–14.0) | 0.98 |
| Monocyte chemoattractant protein-1 (pg/mL)^f^ | 156.2 (116.8–209.8) | 158.5 (128.4–201.9) | 171.4 (135.0–224.2) | 0.26 |
| A proliferation-inducing ligand (pg/mL)^f^ | 541.4 (380.5–798.2) | 647.0 (462.7–925.7) | 604.2 (426.9–926.2) | 0.19 |
| B cell-activating factor (pg/mL)^f^ | 893.5 (694.6–1079.3) | 864.8 (737.6–1100.9) | 862.4 (708.2–1039.3) | 0.68 |

Data are expressed as numbers (%) or median (interquartile range).

^a,b^Proportions were calculated in a row-wise manner.

^a^The patients were stratified into three groups based on HS and GS according to the Asian Working Group for Sarcopenia 2019 criteria [1].

^b^The patients were equally stratified into tertiles according to three component summary scores calculated from KDQOL-SF questionnaire: the score ranges for PCS were 17.5–38.6 (low), 38.7–48.5 (medium), and 48.6–61.7 (high); the score ranges for MCS were 9.4–38.4 (low), 38.5–48.3 (medium), and 48.4–68.7 (high); and the score ranges for KDCS were 24.8–61.9 (low), 62.0–75.2 (medium), and 75.3–97.4 (high).

^c^weight gain (*n* = 54), stable (*n* = 116), and weight loss (*n* = 68).

^d^weight gain (*n* = 36), stable (*n* = 55), and weight loss (*n* = 40).

^e^weight gain (*n* = 51), stable (*n* = 125), and weight loss (*n* = 86).

^f^weight gain (*n* = 47), stable (*n* = 123), and weight loss (*n* = 78).

Abbreviations: KDQOL-SF, kidney disease quality of life short form; HS, handgrip strength; GS, gait speed.

**Table S3. Hazard ratios of covariables**

**a**. Multivariable-adjusted Cox proportional-hazards model for all-cause mortality

| All-cause mortality | Multivariable analysis | |
| --- | --- | --- |
|  | HR (95% CI) | *p* value |
| Sex (male as a reference) | 0.77 (0.46–1.29) | 0.32 |
| Age (per 10 years) | 1.71 (1.39–2.10) | 3.41E-07 |
| Diabetes mellitus | 2.15 (1.35–3.42) | 0.001 |
| Hemoglobin (g/dL) | 0.79 (0.60–1.04) | 0.09 |
| Albumin (g/dL) | 0.26 (0.12–0.57) | 0.0006 |
| Single-pool Kt/V | 0.88 (0.33–2.36) | 0.81 |
| Hemodialysis modality (hemodialysis as a reference) | 1.24 (0.74–2.07) | 0.41 |
| Dialysis vintage (year) | 1.04 (1.00–1.07) | 0.04 |
| Blood flow (per 10 mL/min) | 0.97 (0.89–1.07) | 0.57 |

**b**. Fine-Gray model for MACE

| All-cause mortality | Multivariable analysis | |
| --- | --- | --- |
|  | HR (95% CI) | *p* value |
| Sex (male as a reference) | 1.48 (0.69–3.16) | 0.31 |
| Age (per 10 years) | 1.50 (1.06–2.13) | 0.02 |
| Diabetes mellitus | 3.11 (1.39–6.97) | 0.006 |
| Hemoglobin | 1.13 (0.70–1.82) | 0.63 |
| Albumin | 0.66 (0.13–3.35) | 0.62 |
| Single-pool Kt/V | 1.07 (0.15–7.67) | 0.95 |
| Hemodialysis modality (hemodialysis as a reference) | 0.40 (0.13–1.22) | 0.11 |
| Dialysis vintage | 1.02 (0.97–1.06) | 0.51 |
| Blood flow (per 10 mL/min) | 1.11 (0.96–1.29) | 0.16 |

**Table S4. Cause-specific hazard ratios for cardiovascular events according to annual dry weight change**

**a**. Hazard ratio for MACE

| MACE | Events/Patients (%) | Events/1,000 person-year | Univariate analysis | |  | Multivariate analysis | |
| --- | --- | --- | --- | --- | --- | --- | --- |
|  |  |  | HR (95% CI) | *p* value |  | HR (95% CI) | *p* value |
| Stable | 10/245 (4.1) | 11.9 | Reference | |  | Reference | |
| Mild and moderate weight gain | 11/112 (9.8) | 31.6 | 2.61 (1.11–6.14) | 0.03 |  | 2.98 (1.24–7.16) | 0.01 |
| Mild and moderate weight loss | 10/168 (6.0) | 18.9 | 1.60 (0.67–3.85) | 0.29 |  | 1.39 (0.57–3.42) | 0.47 |

**b**. Hazard ratio for MACE plus stable angina with PCI or CABG

| MACE plus stable angina with PCI or CABG | Events/Patients (%) | Events/1,000 person-year | Univariate analysis | |  | Multivariate analysis | |
| --- | --- | --- | --- | --- | --- | --- | --- |
|  |  |  | HR (95% CI) | *p* value |  | HR (95% CI) | *p* value |
| Stable | 21/245 (8.6) | 26.3 | Reference | |  | Reference | |
| Mild and moderate weight gain | 17/112 (15.2) | 52.1 | 1.96 (1.03–3.71) | 0.04 |  | 2.09 (1.09–4.03) | 0.03 |
| Mild and moderate weight loss | 20/168 (11.9) | 39.7 | 1.53 (0.83–2.82) | 0.17 |  | 1.35 (0.71–2.53) | 0.36 |

Abbreviations: MACE, major adverse cardiovascular events; HR, hazard ratio; CI, confidence interval; PCI, percutaneous coronary intervention; CABG, coronary artery bypass graft.

**Table S5. BMI at baseline and after 1 year stratified by baseline BMI**

|  | annual dry weight change | BMI at baseline (kg/m^2^) | BMI after 1 year (kg/m^2^) |
| --- | --- | --- | --- |
| Normal | Stable | 20.8 ± 1.3 | 20.8 ± 1.3 |
|  | Mild weight gain | 21.0 ± 1.1 | 21.9 ± 1.2 |
|  | Moderate weight gain | 20.1 ± 1.0 | 22.5 ± 1.4 |
|  | Mild weight loss | 21.2 ± 1.2 | 20.5 ± 1.2 |
|  | Moderate weight loss | 21.1 ± 1.1 | 19.4 ± 1.0 |
| Overweight | Stable | 23.9 ± 0.6 | 23.9 ± 0.7 |
|  | Mild weight gain | 23.6 ± 0.5 | 24.7 ± 0.8 |
|  | Moderate weight gain | 24.3 ± 0.5 | 27.0 ± 1.5 |
|  | Mild weight loss | 23.8 ± 0.7 | 23.0 ± 0.7 |
|  | Moderate weight loss | 23.9 ± 0.5 | 21.8 ± 0.5 |
| Obese | Stable | 28.6 ± 3.5 | 28.6 ± 3.5 |
|  | Mild weight gain | 28.0 ± 3.0 | 28.9 ± 3.1 |
|  | Moderate weight gain | 31.6 ± 7.2 | 35.2 ± 8.5 |
|  | Mild weight loss | 28.5 ± 4.0 | 27.5 ± 3.8 |
|  | Moderate weight loss | 27.9 ± 3.7 | 25.1 ± 4.2 |

Abbreviations: BMI, body mass index.

**Table S6. BMI at baseline and after 1 year stratified by average serum albumin levels**

|  | annual dry weight change | BMI at baseline (kg/m^2^) | BMI after 1 year (kg/m^2^) |
| --- | --- | --- | --- |
| Average albumin <3.5 g/dL | Stable | 25.1 ± 5.2 | 25.1 ± 5.1 |
|  | Mild weight gain | 23.9 ± 4.1 | 24.9 ± 4.1 |
|  | Moderate weight gain | not applicable | |
|  | Mild weight loss | 19.6 ± 2.8 | 19.0 ± 2.7 |
|  | Moderate weight loss | 22.5 ± 3.6 | 19.7 ± 3.2 |
| Average albumin ≥3.5 g/dL | Stable | 23.2 ± 4.0 | 23.2 ± 4.0 |
|  | Mild weight gain | 22.8 ± 3.4 | 23.7 ± 3.5 |
|  | Moderate weight gain | 23.2 ± 6.5 | 26.0 ± 7.2 |
|  | Mild weight loss | 23.2 ± 4.0 | 22.4 ± 3.9 |
|  | Moderate weight loss | 24.0 ± 4.4 | 22.0 ± 4.0 |

Abbreviations: BMI, body mass index.

**Table S7. BMI at baseline and after 1 year stratified by age**

|  | annual dry weight change | BMI at baseline (kg/m^2^) | BMI after 1 year (kg/m^2^) |
| --- | --- | --- | --- |
| Age <65 years | Stable | 23.2 ± 4.3 | 23.3 ± 4.4 |
|  | Mild weight gain | 23.1 ± 3.8 | 24.0 ± 3.9 |
|  | Moderate weight gain | 23.4 ± 8.2 | 26.3 ± 9.1 |
|  | Mild weight loss | 23.1 ± 5.1 | 22.4 ± 4.9 |
|  | Moderate weight loss | 23.9 ± 3.8 | 22.1 ± 3.5 |
| Age ≥65 years | Stable | 23.5 ± 3.6 | 23.5 ± 3.6 |
|  | Mild weight gain | 22.6 ± 2.4 | 23.5 ± 2.4 |
|  | Moderate weight gain | 23.0 ± 2.8 | 25.6 ± 3.3 |
|  | Mild weight loss | 22.8 ± 2.2 | 22.0 ± 2.2 |
|  | Moderate weight loss | 23.5 ± 4.7 | 21.1 ± 4.4 |

Abbreviations: BMI, body mass index.

**Figure S1. Distribution of annual dry weight change in the study population**

**Figure S2. Forest plot presenting hazard ratios for all-cause mortality and MACE according to baseline body mass index**

**
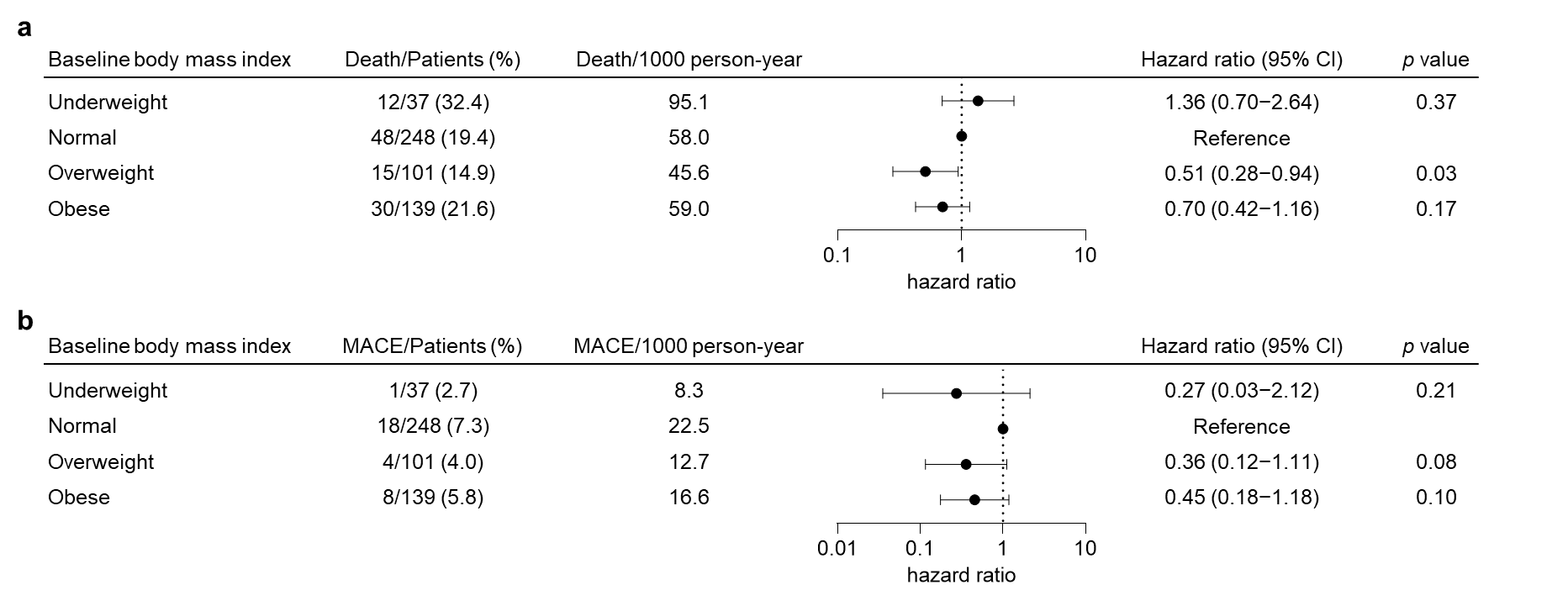
**

The study outcomes included all-cause mortality (a) and major adverse cardiovascular events (b). Hazard ratios and confidence intervals were estimated using Cox proportional hazards models with adjustment for age, sex, diabetes mellitus, average hemoglobin, average serum albumin, single-pool Kt/V, hemodialysis modality (conventional hemodialysis versus hemodiafiltration), dialysis blood flow, and dialysis vintage. The closed circle represents the hazard ratio, and the horizontal line indicates the 95% confidence interval.

**Reference**

1. Chen LK, Woo J, Assantachai P, Auyeung TW, Chou MY, Iijima K, et al. Asian working Group for sarcopenia: 2019 Consensus update on sarcopenia diagnosis and treatment. J. Am. Med. Dir. Assoc. 2020;21:300–307.e2.
